# A Drosophila platform identifies a novel, personalized therapy for a patient with adenoid cystic carcinoma

**DOI:** 10.1016/j.isci.2021.102212

**Published:** 2021-02-20

**Authors:** Erdem Bangi, Peter Smibert, Andrew V. Uzilov, Alexander G. Teague, Sindhura Gopinath, Yevgeniy Antipin, Rong Chen, Chana Hecht, Nelson Gruszczynski, Wesley J. Yon, Denis Malyshev, Denise Laspina, Isaiah Selkridge, Huan Wang, Jorge Gomez, John Mascarenhas, Aye S. Moe, Chun Yee Lau, Patricia Taik, Chetanya Pandya, Max Sung, Sara Kim, Kendra Yum, Robert Sebra, Michael Donovan, Krzysztof Misiukiewicz, Celina Ang, Eric E. Schadt, Marshall R. Posner, Ross L. Cagan

**Affiliations:** 1 Department of Cell, Development, and Regenerative Biology, Icahn School of Medicine at Mount Sinai, New York, NY 10029, USA; 2 Department of Medicine, Hematology and Medical Oncology, Icahn School of Medicine at Mount Sinai, New York, NY 10029, USA; 3 Department of Genetics and Genomic Sciences and Icahn Institute for Genomics and Multiscale Biology, Icahn School of Medicine at Mount Sinai, New York, NY 10029, USA; 4 Sema4, Stamford, CT 06902, USA; 5 Department of Pharmacy, The Mount Sinai Hospital, New York, NY 10029, USA; 6 Department of Pathology, Icahn School of Medicine at Mount Sinai, New York, NY 10029, USA; 7 Tisch Cancer Institute, Icahn School of Medicine at Mount Sinai, New York, NY 10029, USA

**Keywords:** molecular physiology, genetics, biotechnology, cancer systems biology

## Abstract

Adenoid cystic carcinoma (ACC) is a rare cancer type that originates in the salivary glands. Tumors commonly invade along nerve tracks in the head and neck, making surgery challenging. Follow-up treatments for recurrence or metastasis including chemotherapy and targeted therapies have shown limited efficacy, emphasizing the need for new therapies. Here, we report a Drosophila-based therapeutic approach for a patient with advanced ACC disease. A patient-specific Drosophila transgenic line was developed to model the five major variants associated with the patient's disease. Robotics-based screening identified a three-drug cocktail—vorinostat, pindolol, tofacitinib—that rescued transgene-mediated lethality in the Drosophila patient-specific line. Patient treatment led to a sustained stabilization and a partial metabolic response of 12 months. Subsequent resistance was associated with new genomic amplifications and deletions. Given the lack of options for patients with ACC, our data suggest that this approach may prove useful for identifying novel therapeutic candidates.

## Highlights

• Personalized therapy was developed for patient with Adenoid Cystic Carcinoma
• Genomics analysis was leveraged to establish a Drosophila ‘personalized patient avatar’
• A robotics-based screen identified a novel three drug therapeutic cocktail
• 12 months response was followed by relapse and significant tumor genomic re-wiring

## Graphical abstract

**Figure:**
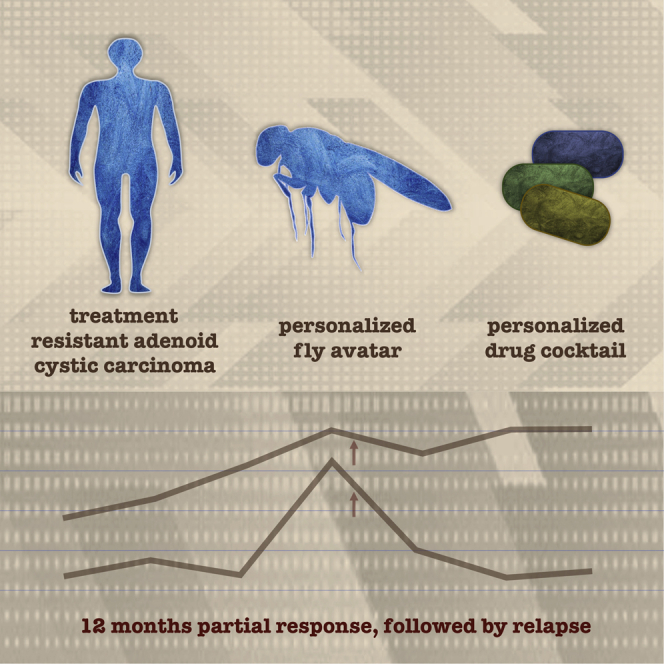

## Introduction

Adenoid cystic carcinoma (ACC) is a relatively rare neoplasm that metastasizes frequently and widely. ACC is the most common malignant tumor of the minor salivary glands and the second most common of the major salivary glands (Coca-Pelaz et al., 2015). Despite early dissemination, it is relatively slow growing. In the United States, approximately 20,000 patients are living with ACC in various stages of progression. One thousand two hundred new cases are reported annually; approximately 60% of those affected are women. On average, patients with ACC present in their 40s and therefore may live with their cancer for decades depending on the rate of progression, with consequent emotional and financial costs to family and society. The median survival is 85% at 5 years and 34% at 15 years, with lymphovascular invasion most associated with poor prognosis (Ouyang et al., 2017).

Patients with ACC have few therapeutic options. Treatment goals are limited and focused on achieving local or regional control through combinations of surgery, radiotherapy, and chemotherapy. Once disseminated or regionally recurrent, there are no effective therapies (Gatta et al., 2020). Chemotherapy and targeted therapies have proven poorly effective with arbitrary and transient responses (Tchekmedyian et al., 2019), while regional, focused therapies such as radiotherapy and surgery are used primarily to reduce symptoms (palliation) to address, *e.g.*, bronchial obstruction and symptomatic bone metastases.

Recent advances in genetic studies have pointed to further challenges: most ACC tumors contain the fusion myelobastosis viral oncogene homolog-nuclear factor 1B (MYB-NF1B (Persson et al., 2009; Andersson and Stenman, 2016) but also include multiple other cancer-associated gene mutations (Ho et al., 2013; Stephens et al., 2013; Ross et al., 2014; Mitani et al., 2016). MYB is a transcriptional activator with a C-terminal inhibitory domain (Sakura et al., 1989; Weston and Bishop, 1989; Dubendorff et al., 1992). Most ACC tumors show activation of MYB through gene fusion of MYB with the transcription factor NFIB due to a 6;9 translocation (Persson et al., 2009; Andersson and Stenman, 2016) or, less often, by truncation or copy number gain (Persson et al., 2012). Fusion or truncation leads to loss of MYB's C-terminus, which is sufficient to generate a constitutively active MYB protein (Gonda et al., 1989); though unlikely (Persson et al., 2009), a function for the accompanying small (5 amino acids) C-terminal fragment of NFIB has not been ruled out. Most patients have additional mutations in other cancer-related genes such as activating mutations in the NOTCH1 and ERBB3 receptors and regulators of signal transduction and cell cycle (Ho et al., 2013; Stephens et al., 2013; Ross et al., 2014; Mitani et al., 2016). Our recent works, both basic and clinical (Bangi et al., 2016, 2019; Levine and Cagan, 2016; Levinson and Cagan, 2016), are consistent with a growing body of work demonstrating that tumor heterogeneity and genetic complexity can lead to drug resistance.

**Figure 1:**
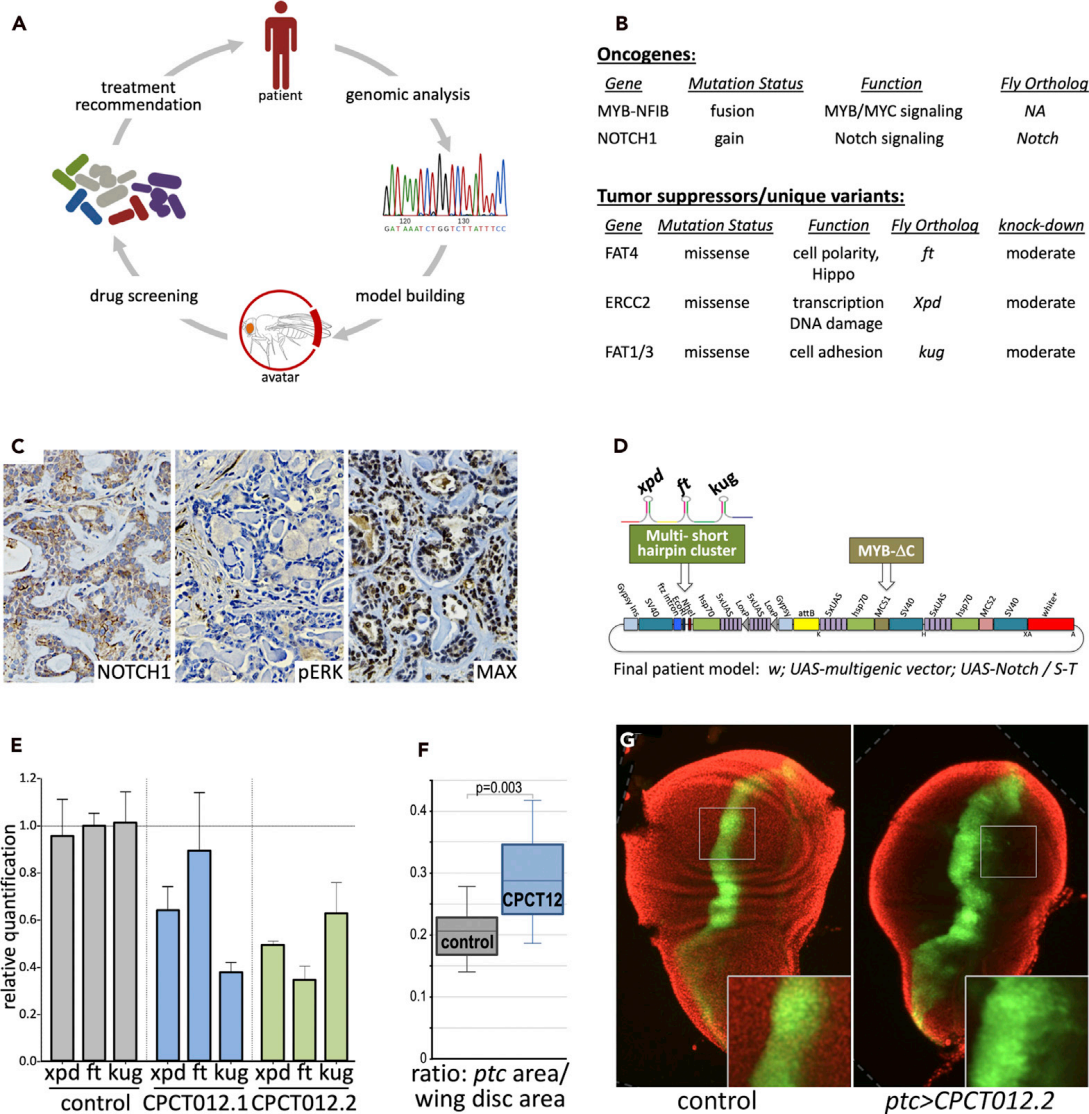
Developing a personalized Drosophila avatar screening platform (A) Overview of personalized approach. Genomic analysis of the patient's tumor identified predicted tumor drivers used to develop a personalized fly avatar. Robotics-based drug screening identified a three-drug cocktail that was vetted for safety by a tumor board and internal review board. (B) Prioritized oncogenes and tumor suppressors that emerged from our genomic analysis. *FAT4*, *ERCC2*, and *FAT1/FAT3* were heterozygous. See also supplemental figure, tables. (C) Immunohistochemistry (brown) identified high levels of plasma membrane and nuclear NOTCH1, indicating elevated NOTCH1 protein and activity in patient tumor sections obtained prior to treatment. Similar immunohistochemical assays failed to validate elevated MAP2K2 activity (pERK) or loss of MAX, and neither were included in the final avatar model. (D) Schematic of transformation vector used to target 4 of 5 cancer genes to different Drosophila tissues. Inducible Notch overexpression (*UAS-Notch*) was introduced by standard genetic crosses. (E) Small hairpins targeting *xpd*, *ft*, and *kug* in CPCT012.2 led to a ~50% reduction in expression as assessed with qPCR. We used this line as the best model of heterozygosity. (F) Quantifying results of directing *ptc > CPCT012* expression on the wing's *ptc* domain, which led to expansion of the domain including a loss of the sharp boundary. Results are represented as the ratio of the *ptc* domain area to total wing disc area. (G) Example of *ptc > CPCT012*-mediated expansion. The *ptc* domain was visualized with an included *UAS-GFP* marker (green). Insets highlight expansion; dotted lines indicate added black background to square images. Error bars represent standard error of the mean.

Recently, we described a personalized fly-to-bedside therapeutic discovery platform (Bangi et al., 2019) (Figure 1A). Modeling the disease of a patient with colorectal cancer in a personalized Drosophila transgenic model, we identified a novel two-drug cocktail that proved effective in both Drosophila and in the modeled patient. Building on this work, we present here a fly-to-bedside platform for ACC, a tumor that has resisted targeted therapies. We developed a personalized Drosophila line that targeted five genes altered in the patient's tumor. This “personalized avatar” exhibited aspects of transformation. We used this line as a screening tool to identify vorinostat-pindolol-tofacitinib as a three-drug cocktail that rescued transgene-mediated lethality in the fly avatar and led to stable disease and a metabolic response in the patient lasting for 12 months.

## Results

### Clinical history

The patient presented with an extensive left-sided maxillary sinus ACC in February, 2013. At the time of presentation, the patient was a 54-year-old Caucasian male with no other significant medical problems. The tumor was found to invade the skin of the face, as well as the base of the skull on imaging. The patient underwent an extensive surgical resection (removal) of gross disease followed by reconstruction of the face and orbit. The tumor was staged as T3N0M0—indicating primarily localized disease—with perineural invasion and pathologically positive post-surgical margins.

The patient was treated with adjuvant proton beam radiation and weekly carboplatin and paclitaxel for 7 weeks for local regional control, completing therapy in June 2013. He underwent periodic surveillance imaging; enlarging pulmonary metastases were identified in March 2015. He was followed expectantly for symptoms and growth with periodic positron emission tomography and X-ray computed tomography (PET/CT) scans.

In January 2016, the patient was consented for participation in the “Personalized Cancer Therapy for Patients With Metastatic Medullary Thyroid or Metastatic Colon Cancer (NCT02363647)” protocol under a rare cancer cohort. A sample was obtained by excisional biopsy of a lung metastasis. This sample confirmed the diagnosis of ACC and was used for genetic analysis and confirmatory studies of genetic findings. Symptomatic bone metastases were identified in February 2017 by positron emission tomography (PET) imaging and magnetic resonance imaging. The patient received radiotherapy in March, May, and October, 2017 to thoracic bone metastases and a scapular metastasis, respectively. Imaging by PET/CT demonstrated increasing size and the number of bone and pulmonary metastases with increasing 2-deoxy-2-[^18^F]fluoro-D-glucose (FDG) uptake during this period of time and prior to starting therapy.

The patient was consented for treatment on the protocol for a personalized treatment plan after reviewing results of drug screening on his tumor-matched fly avatar line. Assessment of his tumor by PET and CT imaging immediately prior to treatment demonstrated continued progression with growth of metastases, new metastases, and a rising standard uptake value (SUV) indicating increased metabolic activity by the tumor.

### Genomic analysis and variant selection

Our overall approach is summarized in Figure 1A. The first step toward building a personalized Drosophila model for the patient was a comprehensive analysis of the tumor genomic landscape. Using a freshly frozen specimen from a lung metastasis obtained from a 2016 specimen prior to treatment on our study, we extracted DNA and RNA as well as DNA from a blood sample as matched control. For genomic analysis of small molecular variants and copy number variants (CNVs), we performed whole-exome sequencing (WES) using tumor and matched normal blood DNA as well as RNA sequencing (RNA-seq).

The ACC genomic landscape is typically diverse and includes many low frequency drivers (Ho et al., 2013; Rettig et al., 2016). As a result, identifying driver alterations and building representative models are challenging. Commonly altered pathways in ACC tumors include overactivation of the MYB/MYC, NOTCH and FGF/IGF/PI3K pathways, as well as alterations in DNA damage repair and chromatin remodeling pathway components (Ho et al., 2013; Rettig et al., 2016). WES of our patient's tumor DNA from the 2016 specimen—obtained prior to treatment—identified 11 nonsynonymous somatic mutations (SNVs/indels) with allelic fraction (AF) ≥0.05 (Table S1), none of which were in genes previously associated with ACC. Most were novel, functionally uncharacterized variants in genes that were not previously associated with cancer.

In the absence of experimental data, we utilized functional prediction algorithms to determine the likelihood that each variant was deleterious (*i.e.* had a negative impact on protein function) (Kircher et al., 2014; Liu et al., 2016). Most WES variants found in the patient's tumor were predicted to be benign by two different functional prediction tools and were eliminated. Finally, we discarded variants that were not detected by RNA-seq, suggesting that they were either false positives or they were not expressed in the tumor. At the end of our analysis, we concluded that none of the somatic variants were appropriate for model building.

In addition to somatic mutations, we also identified 935 rare germline variants in the patient's non-tumor (*i.e.* blood) DNA. Given this large number, we focused our analysis on variants in genes previously associated with cancer, as well as those encoding components of cancer relevant pathways and cellular processes. Of these, we found that heterozygous missense mutations in four genes—*FAT4*, *FAT1*, *FAT3*, and *ERCC2*—were predicted to be deleterious and also detected by tumor RNA sequence data, indicating that the mutant alleles were expressed in the tumor. These were selected for the fly model (Figure 1B).

NOTCH1 is a component of the NOTCH signaling pathway frequently activated in ACC (Ho et al., 2013). Although copy number analysis was inconclusive, elevated NOTCH1 protein in the tumor specimen was established by immunohistochemistry (Figure 1C). Two other potential variants, MAP2K2 (predicted gain) and MAX (predicted loss), were rejected by similar immunohistochemical criteria (Figure 1C). In addition, RNA-seq data revealed a t(6;9) (q22-23;p23-24) MYB-NFIB fusion event in our patient's tumor, a commonly observed cancer driver in patients with ACC (Ho et al., 2019). In our patient's tumor, the fusion event resulted in an out-of-frame transcript encoding a truncated, constitutively active MYB protein that lacked the C-terminal cytoplasmic domain required to regulate its activity (West et al., 2011). NOTCH1 and truncated MYB—which together define a common subtype of adenoid cystic carcinoma (Ho et al., 2019)—were also selected for the fly model, bringing the final number of modeled cancer drivers to six patient variants modeled by five targeted fly genes (Figure 1B).

### Model building and validation

To create a patient-specific model that represents the six alterations identified in our genomic analysis (Figure 1B), we utilized a multigenic vector platform that we tailored for this purpose (Bangi et al., 2019). This vector carries three different multiple cloning sites, each flanked by promoter and transcription terminator sequences: two are designed for protein expression to model oncogenes, and one is reserved for short hairpin-mediated knockdown of Drosophila tumor suppressor orthologs (Figure 1D). Transgenes were cloned downstream of a GAL4-inducible *UAS* promoter, a well-established ectopic expression system in Drosophila that allows both spatial and temporal control of transgene expression (Brand and Perrimon, 1993). Transgene expression was targeted by crossing the patient-specific transgenic line to transgenic fly lines that express GAL4 in specific tissues, such as *ptc-GAL4*. The result was a “*ptc > CPCT012.2*” transgenic fly line that expressed the transgenes—targeting five fly orthologs of six patient variants—in discrete regions across the developing fly.

Previous work found that expressing full-length c-MYB in Drosophila can disrupt developing tissues including aspects of the cell cycle (Lipsick et al., 2001; Fitzpatrick et al., 2002; Davidson et al., 2005). To model the *MYB-NFIB* fusion, we generated a truncated *MYB* construct that represents the product of the translocation event (*MYBΔC*). *NOTCH1* copy gain was modeled by overexpressing a wild-type Drosophila Notch cDNA. Heterozygous missense variants in *FAT4*, *FAT1/3*, and *ERCC2* were modeled by targeting their Drosophila orthologs using short hairpins designed to achieve moderate knockdown (to model the heterozygous nature of each variant). Individual hairpins targeting each gene were selected using previously reported protocols (Vert et al., 2006; Ni et al., 2011) and stitched together as a synthetic multi-hairpin cluster using our microRNA-inspired design (Bangi et al., 2019). As hairpin selection relies on algorithms to predict efficacy (Vert et al., 2006; Ni et al., 2011), we generated two hairpin clusters targeting the same three Drosophila genes with different hairpins—*12.1* and *12.2*—to increase the likelihood of success.

To build the patient-specific multigenic vector, the *MYBΔC* coding sequence and the hairpin cluster were cloned into their respective multiple cloning sites, each downstream of their own inducible UAS promoter (Figure 1D). We generated two different versions of the patient model: CPCT012.1 and CPCT012.2. Both versions carried the same MYBΔC transgene but a different hairpin cluster designed to reduce expression of Drosophila orthologs of *FAT4*, *FAT1/3*, and *ERCC2*. We established two transgenic lines using a site-specific chromosomal integration method mediated by standard ɸC31-based integration (Bischof et al., 2007). Once transgenic lines were established, an existing *Notch* transgenic construct that expresses the full-length Drosophila Notch protein under *UAS* control (Matsuno et al., 2002) was introduced into each line by standard genetic crosses.

Once the two final patient models were established, we ubiquitously expressed each multigenic construct (*tub > CPCT012*) in developing larvae to determine whether the hairpin clusters we generated were effective. Quantitative polymerase chain reaction (qPCR) analysis indicated that the short hairpin cluster in CPCT012.2 was effective in moderately reducing expression of all three genes, our goal for modeling heterozygous variants; CPCT012.1 did not show significant knockdown of the Drosophila FAT4 ortholog *ft* (Figure 1E). We therefore focused on the CPT012.2 patient-specific transgenic line for further characterization.

To further validate the CPCT012.2 line, we used *ptc-GAL4* to direct transgene expression within several tissues including a discrete stripe of expression at the developing wing epithelium's anterior/posterior boundary (Figures 1F and 1G). In previous work, expressing oncogenes with the *ptc-GAL4* driver led to expansion of the *ptc* domain in the developing wing, reflecting overproliferation (Vidal et al., 2006; Vidal et al., 2007; Levinson and Cagan, 2016; Sonoshita et al., 2018). Consistent with promoting at least one aspect of transformation, expressing the transgenes at the larval wing boundary (*ptc > CPCT012.2*) led to expansion of the *ptc* domain (Figures 1F and 1G). We concluded this model could prove useful as an accessible whole animal platform to screen for candidate therapeutics.

### Drug screening

Rescue from lethality is a useful primary readout for whole animal drug screens, providing rapid, quantitative data (Rudrapatna et al., 2014; Levine and Cagan, 2016; Bangi et al., 2019). We have successfully used *ptc-gal4* in previous genetic and drug screens of Drosophila cancer models (Dar et al., 2012; Sonoshita et al., 2018). We therefore calibrated *ptc > CPCT012.2* flies for lethality, using temperature to alter GAL4 activity to a level of near-complete animal lethality.

**Figure 2:**
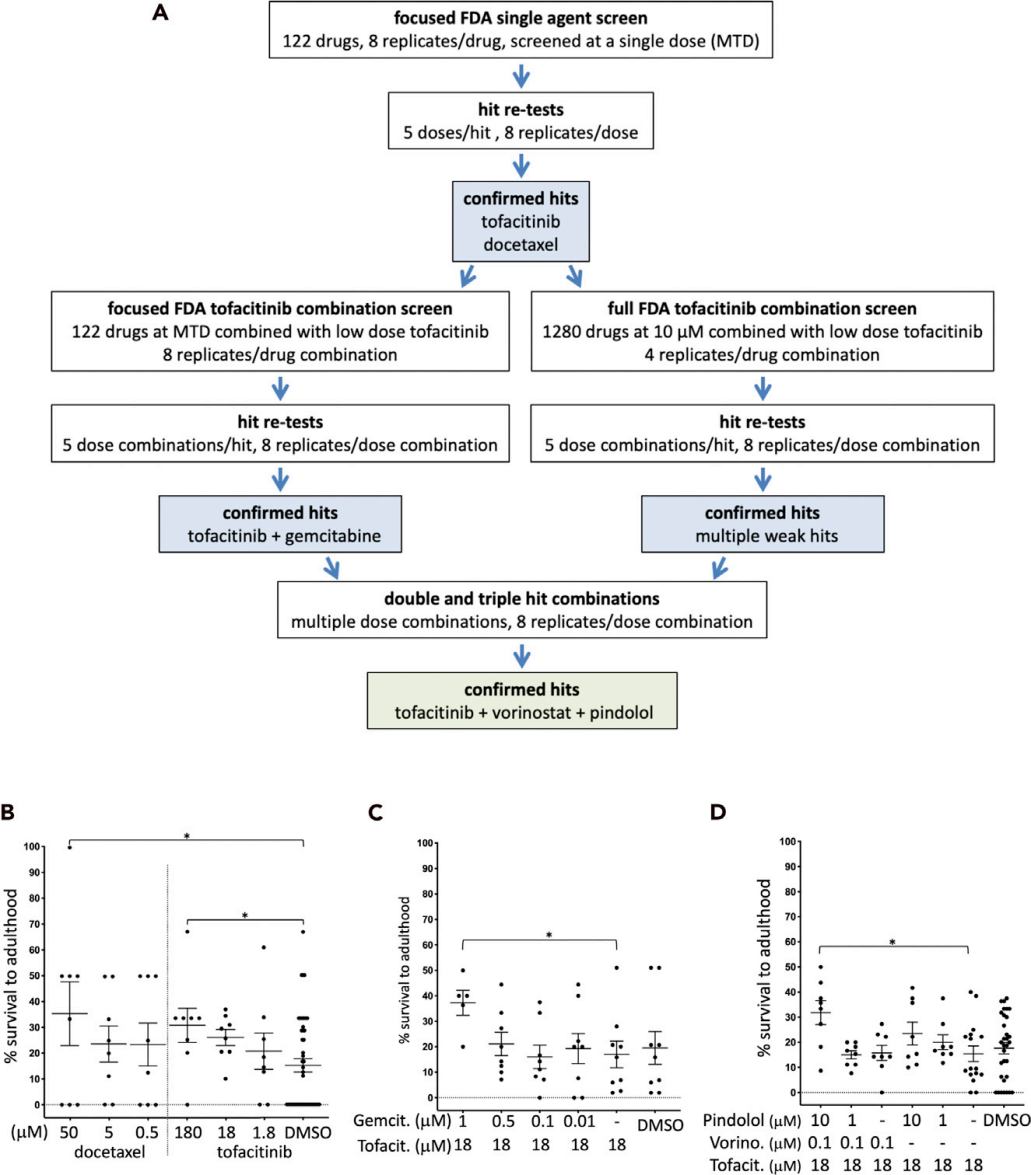
Screen for candidate combinations of FDA-approved drugs (A) Flowchart of multi-step drug screen. An initial screen of the Focused FDA Library yielded tofacitinib and docetaxel as weak single agent hits. Subsequent screens identified tofacitinib, vorinostat, and pindolol as an effective 3-drug combination. (B) Data demonstrating initial rescue by docetaxel and tofacitinib as single agents. (C) Data demonstrating CPCT012 rescue to adulthood by gemcitabine plus tofacitinib, an effective two-drug combination. Tofacitinib was used at a dose below that required for significant rescue. (D) Data demonstrating CPCT012 rescue to adulthood by tofacitinib, vorinostat, and pindolol. The 3-drug combination proved the most effective at rescuing CPCT012 to adulthood. Asterisks (∗) in panels (B–D) indicate p < 0.05 as assessed by Student's t-test. Error bars represent standard error of the mean.

We previously found that most drugs are not effective as single agents against genetically complex cancer models (Bangi et al., 2019). We therefore used an iterative screening process to progressively identify drug combinations (Figure 2A). We first screened a custom-built 'Focused FDA-Approved Library' of 122 drugs/drug combinations enriched for cancer relevant activities. We identified and confirmed two hits with weak efficacy: the chemotherapy drug docetaxel is an anti-microtubule agent; the JAK inhibitor tofacitinib (Changelian et al., 2003; Vyas et al., 2013) is FDA approved for rheumatoid arthritis, ulcerative colitis, and psoriatic arthritis (Figures 2A and 2B). The patient was previously treated with the anti-microtubule agent paclitaxel as part of a combination therapy with carboplatin, and we therefore did not pursue docetaxel further.

Tofacitinib is a promising candidate for combination screens. Its target pathway, JAK/STAT signaling, can be activated by NOTCH in cancer cells (Jin et al., 2013), which was elevated in the patient's tumor. Re-screening the Focused Library in the presence of low dose tofacitinib (see methods) identified the DNA synthesis inhibitor and FDA-approved chemotherapy agent gemcitabine as an effective partner for tofacitinib (Figures 2A and 2C). We also screened a commercially available “FDA-Approved Drug Library” of 1280 drugs approved for all indications, again in combination with low dose tofacitinib. These combination screens identified several tofacitinib drug combinations with low efficacy. We then tested all confirmed hits in double and triple combinations (Figure 2A). From these screens, a three-drug cocktail emerged (Figure 2D): tofacitinib, vorinostat (histone deacetylase inhibitor, anti-cancer agent), and pindolol (a non-selective beta blocker used to treat high blood pressure).

Each drug identified in our screens has been reported to have targets or anti-tumor effects that could be relevant to our patient tumor's genomic profile. Tofacitinib is an inhibitor of JAK/STAT signaling (Vyas et al., 2013), a cancer-relevant pathway that can be activated downstream of Notch signaling in tumor cells (Jin et al., 2013). Gemcitabine is a nucleoside analog that interferes with DNA synthesis (Plunkett et al., 1995) and is approved for treatment of multiple cancer types. The histone deacetylase (HDAC) inhibitor vorinostat may be particularly relevant for treatment of ACC, as deregulation of chromatin remodeling is observed in about 35% of sequenced tumors (Duvic and Vu, 2007; Marks and Breslow, 2007; Ho et al., 2013). A mechanism of action clearly relevant to cancer has not been reported for pindolol. However, there is some evidence suggesting that inhibition of β-adrenergic signaling by beta blockers can have anti-tumor effects, including inhibition of tumor cell proliferation, migration, invasion, angiogenesis, and metastases (Creed et al., 2015; Partecke et al., 2016; Wrobel et al., 2016). A positive correlation between beta blocker use and cancer-specific survival has been documented (Na et al., 2018), although no causal relationship between the two has been reported.

Our findings were reviewed by a multidisciplinary tumor board that included pharmacists and oncologists with expertise in clinical trial design and dosing. The tumor board raised some concerns regarding myelosuppression, the major dose limiting toxicity associated with gemcitabine. Given the concerns with gemcitabine and both the clinical relevance of the signaling nodes targeted by the triple drug combination and their particular importance for this patient's tumor genome landscape, the tumor board unanimously selected the tofacitinib/vorinostat/pindolol triple combination as the first line recommendation for the patient.

### Patient treatment

The patient initiated treatment orally with 400 mg of vorinostat daily (2800 mg/week), 10 mg of tofacitinib daily, and 10 mg of pindolol daily beginning on 4/19/18. At four weeks, grade 1 thrombocytopenia, creatinine elevation, and folliculitis were observed likely due to vorinostat; minor fatigue was reported likely due to pindolol. All the drugs were held and then restarted 10 days later with 400 mg of vorinostat reduced to five days per week (2000 mg/week). Recurrent rash, thrombocytopenia, and creatinine elevation developed after restarting vorinostat, which resolved after halting and then restarting vorinostat with a further dose reduction to 300 mg four days per week (1200 mg/week) after which creatine and platelet counts remained within normal limits.

**Figure 3:**
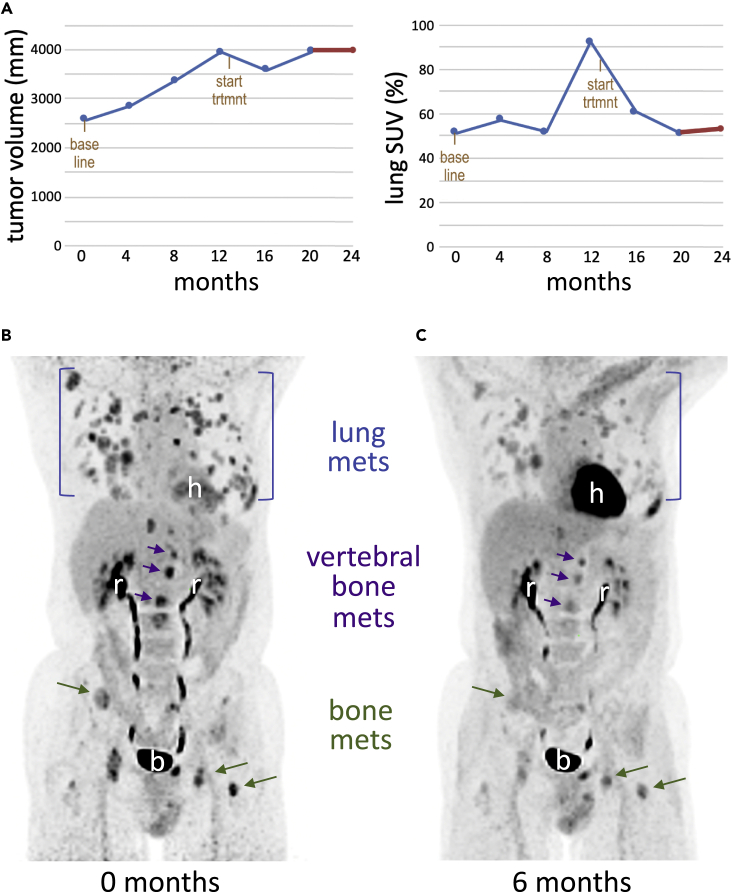
Body PET scans from baseline and after 6 months of treatment (A) Prior to the start of treatment, tumor volume (*upper* panel) and standardized uptake value (SUV; *lower* panel) of the 2-deoxy-2-[^18^F]fluoro-D-glucose (FDG) tracer were increasing over time, indicating progressive disease. Initiation of treatment led to stabilization of total tumor volume and reduction of lung SUV. (B) Control scans just prior to treatment highlight extensive tumor metastases in the bone and lung. (C) Glucose tracer (FDG) uptake in the lung and bone metastases was substantially reduced after 6 months of therapy in imaged sites; further, no new lesions appeared. These data indicate clinical benefit from the drug treatment, manifested as reduced FDG uptake and absence of progression. h = heart, r = renal tubules, b = bladder.

In response to treatment, the patient exhibited documented stable disease (Figure 3) with no new bone lesions and mild regression of lung lesions. Quantitative imaging of a FDG tracer with PET/CT scans was used to measure relative glucose uptake by the tumor. Cumulative FDG data over time indicated a significant reduction (49%) in SUV in pulmonary and bone metastases (Figure 3), indicating a significant metabolic response.

The patient continued on therapy until 4/19/2019 (12 months), when he exhibited documented progression by progression by response evaluation criteria in solid tumors (RECIST), version 1.1, criteria. He had subsequent palliative surgery, chemotherapy, and radiation for further rapid progression. The patient passed away on 3/17/2020.

### Post-treatment analysis

**Figure 4:**
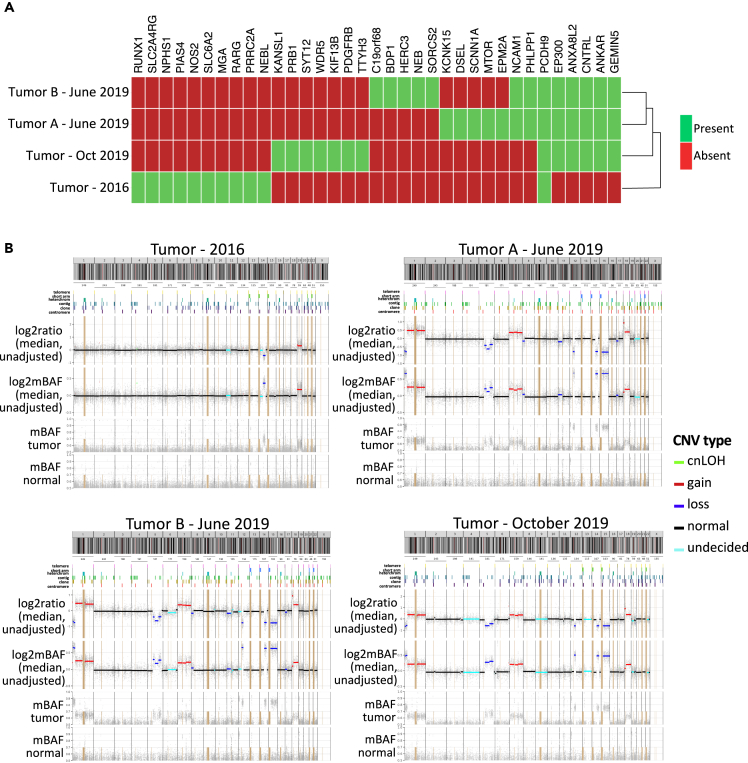
Patient somatic genomic profiles The patient tumor samples from 2016 to 2019 exhibited significant genomic differences. (A and B) (A) Somatic protein-altering molecular variants (SNVs and indels with AF ≥ 0.05) and (B) somatic copy number variant (sCNV) profiles of the four tumor samples are summarized, as assessed with saasCNV (Zhang and Hao, 2015). The 2019 specimens contained *de novo* variants and more unstable sCNV profiles.

To better understand the nature of the emergent resistance at 12 months, we obtained two additional biopsy samples from the patient shortly after he stopped receiving the drug cocktail treatment in April 2019. Similar to the original sample, WES was performed on both biopsies: two specimens were obtained from the same formalin-fixed paraffin-embedded (FFPE) tumor block in June 2019 biopsy; a bone biopsy specimen was obtained in October 2019. The somatic non-synonymous small molecular variants with AF ≥0.05 and CNVs from the post-treatment 2019 specimens were compared to the original pre-treatment 2016 specimen. We observed significant genomic differences (Figure 4).

A small number of somatic variants were common to all 2019 specimens but not the 2016 specimen (Figure 4A and Table S1). These included variants in genes encoding ANKAR, ANXA8L2, CNTRL, EP300, GEMIN5, which are therefore candidates to play a role in the resistance that emerged during treatment. Somatic copy number variants (sCNVs), identified by saasCNV (Zhang and Hao, 2015), found that all three 2019 specimens had a significantly larger number of sCNVs than the original 2016 sample, which had relatively few sCNVs (Figure 4B). Loss of a large segment on chromosome 14 in the original specimen was the sole sCNV retained in 2019 specimens; in contrast, *e.g.*, an aneuploid gain of chromosome 19 from the original specimen was lost in all 2019 specimens, suggesting the aneuploid gain was contained within a subclonal alteration that was selected against as the patient's disease advanced. GATK4 sCNV was used to confirm the sCNV calling in the 2019 specimens (Figure S1, Tables S2 and S3; see also methods); the sCNV profiles between the two callers largely agreed with each other.

Finally, to further identify driver genes responsible for emergent resistance, annotated tumor suppressor genes by Catalogue of Somatic Mutations in Cancer (COSMIC) Cancer Gene Census (Sondka et al., 2018) were checked against the biallelic inactivated genes (defined by the intersection between loss sCNVs and germline protein-altering variants with gnomAD allele frequency ≤ 0.05%). No additional examples of functionally significant variants were found unique to all three 2019 specimens vs. the 2016 specimen (Table S1). Overall, candidates for emergent drug resistance include multiple somatic mutated loci plus significant changes in the CNV landscape.

## Discussion

ACC has proven to be a challenging disease, with limited overall response to traditional and targeted therapies. We therefore undertook an experimental approach based on efforts to model its genomic complexity in the context of a personalized approach. Here, we report our results treating a patient with progressive disease that failed to respond to standard-of-care treatments: a novel three-drug cocktail provided stable disease for 12 months, followed by treatment resistance and extensive genomic alterations observed in biopsy samples.

Building on an approach we previously reported for a patient with advanced colorectal cancer adenocarcinoma (Bangi et al., 2019), we used a decision tree approach to prioritize six genes that were identified as key for affecting tumor progression and, potentially, drug response. Additional tools such immunohistochemistry (Figure 1) were used to help validate our gene choices, but especially given the presence of CNVs and the timeline required for patient treatment, our analysis is necessarily incomplete.

ACC is commonly associated with truncation of MYB and elevated NOTCH1 expression/activity, and this patient's tumor presented with both. ACC tumors are not associated with large numbers of somatic mutations (Ho et al., 2013) and, again, this patient's tumor reflected this as we observed few other somatic changes including CNVs. Germline mutations are often not accounted for in similar analyses. Nevertheless, our germline analysis identified four genes with known functions likely to contribute to tumor progression and potentially drug response: *FAT1* and *FAT3*, *FAT4*, and *ERCC2*, modeled by targeting *kug, ft*, and *xpd*, respectively.

FAT1, FAT3, and FAT4 are atypical cadherins and key regulators of cancer-relevant cellular processes including planar cell polarity and MST/HIPPO signaling, which regulates organ size. They are associated as tumor suppressors in a variety of tumors including ovarian, medullary thyroid, gastric, cervical, colorectal, bladder, and squamous cell carcinomas, as well as ACC (Ho et al., 2013; Gao et al., 2014; Chen et al., 2019; Huang et al., 2019; Skuja et al., 2019; Malgundkar et al., 2020; Melis et al., 2020; Qu et al., 2020; Wang et al., 2020). Reducing these atypical cadherins by approximately 50% expression was therefore considered potentially impactful to our drug screening platform. *ERCC2* encodes XPD, a protein associated with regulation of TFIIH-mediated transcription and DNA damage. XPD has effects on progression of a broad palette of tumor types when mutated or altered—increased or decreased—in expression, and variants in ERCC2 have been associated with altered drug response, especially platinum-based therapies (Du et al., 2014; Fu et al., 2017; Tan et al., 2017; Pajuelo-Lozano et al., 2018; Liu et al., 2019). This may have contributed to the patient's failure to respond to earlier carboplatin-based therapy.

A key advantage of our approach is the ability to identify novel drug combinations selected solely on the basis of efficacy in a whole animal model. The three-drug cocktail—tofacitinib, pindolol, and vorinostat—is especially interesting to consider from a mechanism standpoint. Vorinostat has been reported to provide some benefit to a subset of patients with ACC (Goncalves et al., 2017), though it did not display activity as a single agent in our screens. The primary hit was tofacitinib, a JAK inhibitor used primarily for rheumatoid arthritis. As part of the JAK/STAT pathway, JAK is a signaling kinase with broad effects on development and disease including cancer (Thomas et al., 2015). JAK is positively regulated by NOTCH signaling activity in multiple contexts (Monsalve et al., 2006; Jin et al., 2013; Ho et al., 2015), suggesting a mechanism for tofacitinib activity.

Finally, our genomic analysis of later biopsies highlights the challenge that selection-based resistance poses, even for a three-drug therapeutic cocktail. Our analysis confirmed retention of the MYB-NFIB fusion; however, the CNV landscape was significantly altered including a number of new CNVs in the post-treatment samples. This provides potential insight into the mechanisms by which ACC has proven recalcitrant to most drug treatments: selection for progressive clones can lead to significant genomic changes that can in turn subvert therapeutic activity. One potential response would have been to follow with treatment of our second-line drug cocktail, tofacitinib-gemcitabine. However, follow-up radiation-based therapy and subsequent rapid patient decline made use of gemcitabine contraindicated.

In conclusion, we present a personalized approach to treating ACC. Genomic analysis followed by construction and screening of a “personalized fly avatar” led to a unique three-drug cocktail that promoted stable disease and reduced tumor metabolic activity for 12 months. To date, in our work, this drug cocktail is unique (data not shown), suggesting at least some specificity for this patient. Assessing the broad utility of this whole animal platform approach—and this specific drug cocktail—for patients with ACC will require a larger study. This personalized approach is adaptable to a broad palette of tumor types and may prove especially useful for rare cancers that do not have a standard-of-care second-line therapy or a clear treatment guidance protocol.

### Limitations of the study

This study is based on a single patient and, given the divergence between species, should be considered a first step in determining whether this Drosophila approach can provide patient benefits that match or exceed other available approaches.

### Resource availability

#### Lead contact

Further information and requests for resources and reagents should be directed to and will be fulfilled by the lead contact, Ross Cagan (Ross.Cagan@glasgow.ac.uk).

#### Materials availability

All Drosophila lines generated in this study are available upon request. All data and accession numbers needed to evaluate the conclusions in the paper are present in the paper and/or the supplemental information. Additional data related to this paper may be requested from the authors.

#### Data and code availability

The published article includes all patient analysis generated during this study except for private genomic data that is restricted due to patient confidentiality.

## Methods

All methods can be found in the accompanying transparent methods supplemental file.

## Supplemental information

Document S1. Transparent methods, Figure S1, and Tables S1–S5
