# Supplementary material for: A Drosophila platform identifies a novel, personalized therapy for a patient with adenoid cystic carcinoma
Source: iScience. 2021 Feb 20;24(3):102212. doi: 10.1016/j.isci.2021.102212 (PMC7940980; doi:10.1016/j.isci.2021.102212)
Supplement: Document S1. Transparent methods, Figure S1, and Tables S1–S5 [file mmc1.pdf]

iScience, Volume *24*

Supplemental information

A Drosophila platform identifies a novel, personalized therapy for a patient with adenoid cystic carcinoma

Erdem Bangi, Peter Smibert, Andrew V. Uzilov, Alexander G. Teague, Sindhura Gopinath, Yevgeniy Antipin, Rong Chen, Chana Hecht, Nelson Gruszczynski, Wesley J. Yon, Denis Malyshev, Denise Laspina, Isaiah Selkridge, Huan Wang, Jorge Gomez, John Mascarenhas, Aye S. Moe, Chun Yee Lau, Patricia Taik, Chetanya Pandya, Max Sung, Sara Kim, Kendra Yum, Robert Sebra, Michael Donovan, Krzysztof Misiukiewicz, Celina Ang, Eric E. Schadt, Marshall R. Posner, and Ross L. Cagan

Supplemental Data

Figure S1

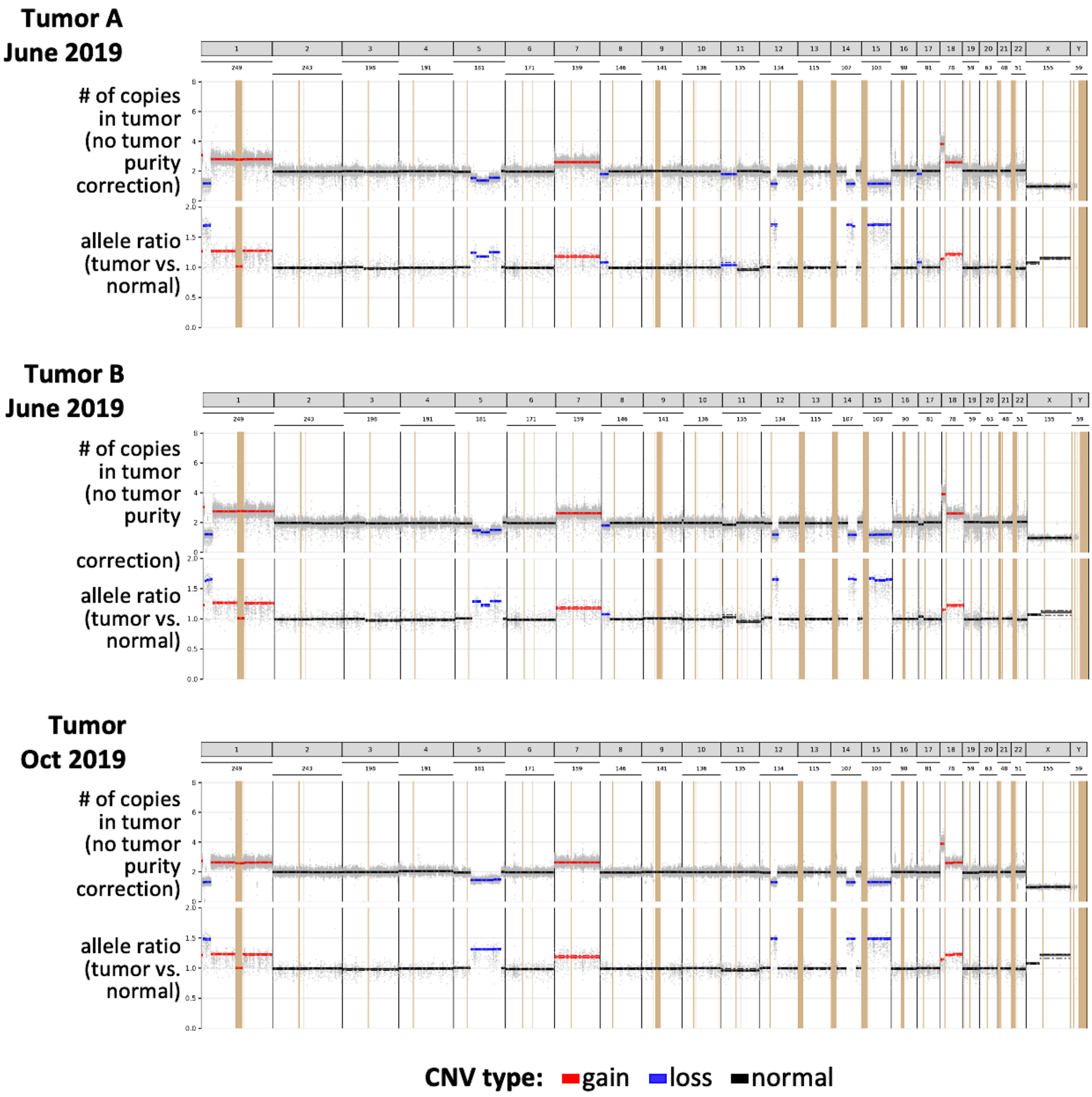

Figure S1: 2019 samples- sCNV profiles; related to Figure 4: GATK4 Somatic CNV algorithm-based profiles of the 2019 specimens are consistent with results from the saasCNV analysis shown in Figure 4.

Table S1

specimen	chrom	pos	ref	alt	AD	Ref_AD	AF	gene_symbol	hgvs_p	effect_seqontology
tumor-2016	6	31600001	G	C	59	172	0.2554	PRRC2A	p.Gly1184Ala	missense_variant
tumor-2016	10	21178863	C	T	49	126	0.28	NEBL	p.Glu57Lys	missense_variant
tumor-2016	12	53605638	C	T	25	67	0.2717	RARG	p.Arg396Lys	missense_variant
tumor-2016	13	67800268	G	A	119	320	0.2711	PCDH9	p.Leu769Phe	missense_variant
tumor-2016	15	42005433	GTA	G	95	253	0.273	MGA	p.Val1057fs	frameshift_variant
tumor-2016	16	55703560	A	G	43	124	0.2575	SLC6A2	p.Asn120Asp	missense_variant
tumor-2016	17	26092584	C	T	8	71	0.1013	NOS2	p.Arg802His	missense_variant
tumor-2016	19	4012931	TGAGTTTTC	T	150	526	0.2219	PIAS4	p.Met13fs	frameshift_variant
tumor-2016	19	36339588	C	T	24	143	0.1437	NPHS1	p.Trp374*	stop_gained
tumor-2016	20	62373487	C	CG	46	144	0.2421	SLC2A4RG	p.Pro195_Ala196fs	frameshift_variant
tumor-2016	21	36231810	C	T	79	227	0.2582	RUNX1	p.Ala165Thr	missense_variant
tumor-A-Jun-2019	1	11227539	G	A	4	65	0.058	MTOR	p.Ala1430Val	missense_variant
tumor-A-Jun-2019	2	190575865	G	T	44	136	0.2444	ANKAR	p.Cys737Phe	missense_variant
tumor-A-Jun-2019	5	154300980	G	A	42	108	0.28	GEMIN5	p.Pro462Leu	missense_variant
tumor-A-Jun-2019	6	146056511	G	GCA	2	12	0.1429	EPM2A	p.Arg41_Pro42fs	frameshift_variant
tumor-A-Jun-2019	9	123902923	A	G	36	68	0.3462	CNTRL	p.Asp781Gly	missense_variant
tumor-A-Jun-2019	9	123902923	A	G	36	68	0.3462	CNTRL	p.Asp229Gly	missense_variant
tumor-A-Jun-2019	10	47756088	C	G	45	131	0.2557	ANXA8L2	p.Ala183Gly	missense_variant
tumor-A-Jun-2019	11	113103461	G	T	32	245	0.1155	NCAM1	p.Asp473Tyr	missense_variant
tumor-A-Jun-2019	12	6458572	CTGT	CACA	8	124	0.0606	SCNN1A	NULL	splice_acceptor_variant
tumor-A-Jun-2019	13	67800268	G	A	129	134	0.4905	PCDH9	p.Leu769Phe	missense_variant
tumor-A-Jun-2019	18	60382986	CCGGCGGCCGCC GCTGCGGCAGCA GCAGCAGCAG CGGCGGCCG	C	3	20	0.1304	PHLPP1	p.Pro24_Ala38del	inframe_deletion
tumor-A-Jun-2019	18	65179718	T	A	26	336	0.0718	DSEL	p.Asn720Tyr	missense_variant
tumor-A-Jun-2019	20	43379283	C	G	5	78	0.0602	KCNK15	p.Pro266Arg	missense_variant
tumor-A-Jun-2019	22	41489055	C	CT	25	185	0.119	EP300	p.Pro16_Lys17fs	frameshift_variant
tumor-B-Jun-2019	2	152521368	TGTA	T	9	121	0.0692	NEB	p.Tyr1749del	inframe_deletion
tumor-B-Jun-2019	2	190575865	G	T	35	141	0.1989	ANKAR	p.Cys737Phe	missense_variant
tumor-B-Jun-2019	4	7194621	A	G	2	6	0.25	SORCS2	p.Asp83Gly	missense_variant
tumor-B-Jun-2019	4	89570991	A	AG	5	91	0.0521	HERC3	p.Glu76_Gln77fs	frameshift_variant
tumor-B-Jun-2019	5	70828195	CCAAA	C	7	88	0.0737	BDP1	p.Pro1945fs	frameshift_variant
tumor-B-Jun-2019	5	154300980	G	A	18	80	0.1837	GEMIN5	p.Pro462Leu	missense_variant
tumor-B-Jun-2019	9	123902923	A	G	39	61	0.39	CNTRL	p.Asp781Gly	missense_variant
tumor-B-Jun-2019	9	123902923	A	G	39	61	0.39	CNTRL	p.Asp229Gly	missense_variant
tumor-B-Jun-2019	10	47756088	C	G	40	101	0.2837	ANXA8L2	p.Ala183Gly	missense_variant
tumor-B-Jun-2019	11	113103461	G	T	14	228	0.0579	NCAM1	p.Asp473Tyr	missense_variant
tumor-B-Jun-2019	13	67800268	G	A	82	152	0.3504	PCDH9	p.Leu769Phe	missense_variant
tumor-B-Jun-2019	18	60382986	CCGGCGGCCGCCGC TGCGGCAGCAGCAG CAGCAGCGGCGGCCG	C	3	18	0.1429	PHLPP1	p.Pro24_Ala38del	inframe_deletion
tumor-B-Jun-2019	19	48698259	GCGT	G	3	28	0.0968	C19orf68	p.Arg313_Val314del	disruptive_inframe_del..
tumor-B-Jun-2019	22	41489055	C	CT	22	165	0.1176	EP300	p.Pro16_Lys17fs	frameshift_variant
tumor-Oct-2019	2	190575865	G	T	82	256	0.2426	ANKAR	p.Cys737Phe	missense_variant
tumor-Oct-2019	5	149505023	C	A	18	287	0.059	PDGFRB	p.Asp598Tyr	missense_variant
tumor-Oct-2019	5	154300980	G	A	27	228	0.1059	GEMIN5	p.Pro462Leu	missense_variant
tumor-Oct-2019	7	2701363	G	GCAGCC	49	504	0.0886	TTYH3	p.Gly521_Ser522fs	frameshift_variant
tumor-Oct-2019	8	28980986	G	A	67	328	0.1696	KIF13B	p.Arg1126Cys	missense_variant
tumor-Oct-2019	9	123902923	A	G	74	142	0.3426	CNTRL	p.Asp781Gly	missense_variant
tumor-Oct-2019	9	123902923	A	G	74	142	0.3426	CNTRL	p.Asp229Gly	missense_variant
tumor-Oct-2019	9	137020477	G	C	43	360	0.1067	WDR5	p.Trp241Cys	missense_variant
tumor-Oct-2019	10	47756088	C	G	95	287	0.2487	ANXA8L2	p.Ala183Gly	missense_variant
tumor-Oct-2019	11	66816217	C	T	34	314	0.0977	SYT12	p.Arg419Trp	missense_variant
tumor-Oct-2019	12	11506922	G	C	216	446	0.3263	PRB1	p.Pro39Ala	missense_variant
tumor-Oct-2019	13	67800268	G	A	169	344	0.3294	PCDH9	p.Leu769Phe	missense_variant
tumor-Oct-2019	17	44110767	A	ACCTCT TCATTCTC CTCATCAGGACTCC CCTTCAGAGACTG	146	228	0.3904	KANSL1	NULL	splice_donor_variant
tumor-Oct-2019	22	41489055	C	CT	29	355	0.0755	EP300	p.Pro16_Lys17fs	frameshift_variant

Table S1: List of variants identified for the four patient tumor samples; related to Figure 1: Note conserved alterations in ANKAR, ANXA8L2, CNTRL, EP300, and GEMIN5 in the 2019 samples. Only the variant in PCDH9 was common to all samples; this was not included in the avatar models because it was not predicted to alter protein function.

Table S2

sample	Call Type	Num Genes	Seg Chrom	segL	segR	Seg Len	Segment Cytoband	Tumor Num Copies	Tumor Num Copies_ciLow	Tumor Num Copies_ciHigh
tumor-Oct-2019	loss	395	chr12	39047434	62261456	23214023	q12q14.1	1.3088	1.3027	1.3143
tumor-Oct-2019	gain	578	chr1	152329836	196716693	44386858	q21.3q31.3	2.6268	2.6182	2.6314
tumor-Oct-2019	loss	270	chr14	54863537	86090091	31226555	q22.2q31.3	1.3071	1.3013	1.3111
tumor-Oct-2019	gain	573	chr1	196857031	249212550	52355520	q31.3q44	2.6372	2.6328	2.6437
tumor-Oct-2019	loss	393	chr1	13182741	34401805	21219065	p36.21p35.1	1.3214	1.3169	1.3257
tumor-Oct-2019	gain	1412	chr7	192950	158937713	158744764	p22.3q36.3	2.6379	2.6327	2.6443
tumor-Oct-2019	gain	126	chr18	18531095	44497558	25966464	q11.1q21.1	2.6021	2.5939	2.6131
tumor-Oct-2019	loss	1013	chr15	20739247	102359139	81619893	q11.2q26.3	1.3155	1.3111	1.3198
tumor-Oct-2019	gain	840	chr1	34497945	120548461	86050517	p35.1p12	2.6399	2.6347	2.6449
tumor-Oct-2019	loss	215	chr5	140865491	167182447	26316957	q31.3q34	1.4964	1.4917	1.501
tumor-Oct-2019	gain	116	chr18	158449	14852729	14694281	p11.32p11.21	3.8927	3.8809	3.9044
tumor-Oct-2019	gain	180	chr1	145414532	152329835	6915304	q21.1q21.3	2.5896	2.5764	2.5998
tumor-Oct-2019	loss	614	chr5	60394569	140865490	80470922	q12.1q31.3	1.4549	1.4524	1.4595
tumor-Oct-2019	gain	185	chr18	44559124	78005481	33446358	q21.1q23	2.6297	2.6192	2.6418
tumor-Oct-2019	loss	125	chr1	5987459	12908010	6920552	p36.31p36.21	1.3051	1.2988	1.3114
tumor-Oct-2019	gain	106	chr1	861072	5969523	5108452	p36.33p36.31	2.7424	2.7338	2.7503
tumor-Oct-2019	gain	42	chr1	120611698	145368934	24757237	p11.2q21.1	2.5783	2.5486	2.6098
tumor-Oct-2019	gain	16	chr14	19377344	20296357	919014	q11.2	3.1031	3.0291	3.2005
tumor-Oct-2019	loss	2	chr1	196743767	196801379	57613	q31.3	1.1489	1.1152	1.183
tumor-Oct-2019	gain	8	chr12	11174021	11286633	112613	p13.2	2.0581	1.9823	2.1423
tumor-Oct-2019	loss	10	chr1	12908011	13001563	93553	p36.21	0.0565	0.0507	0.0639
tumor-Oct-2019	loss	4	chr18	44554323	44555322	1000	q21.1	0.0014	0.0012	0.0015
tumor-Oct-2019	loss	2	chrX	47917607	47920574	2968	p11.23	0.0064	0.0059	0.0068
tumor-Oct-2019	loss	1	chr2	131413872	131415700	1829	q21.1	0.0115	0.0105	0.0125
tumor-A-Jun-2019	loss	395	chr12	39047434	62261456	23214023	q12q14.1	1.1526	1.1475	1.1561
tumor-A-Jun-2019	gain	1331	chr1	145414532	249212550	103798019	q21.1q44	2.8002	2.7877	2.8078
tumor-A-Jun-2019	loss	179	chr14	54863537	74194477	19330941	q22.2q24.3	1.1513	1.1466	1.1581
tumor-A-Jun-2019	loss	393	chr1	13182741	34401805	21219065	p36.21p35.1	1.1775	1.1715	1.1859
tumor-A-Jun-2019	gain	1412	chr7	192950	158937713	158744764	p22.3q36.3	2.6028	2.5908	2.6117
tumor-A-Jun-2019	gain	314	chr18	18531095	78005481	59474387	q11.1q23	2.5895	2.5789	2.5976
tumor-A-Jun-2019	loss	669	chr15	42985064	102359139	59374076	q15.2q26.3	1.1594	1.1546	1.1697
tumor-A-Jun-2019	gain	848	chr1	34497945	121116297	86618353	p35.1p11.2	2.8008	2.7886	2.8145
tumor-A-Jun-2019	loss	441	chr5	123974576	162945619	38971044	q23.2q34	1.5554	1.5481	1.5595
tumor-A-Jun-2019	loss	345	chr15	20739247	42977063	22237817	q11.2q15.2	1.1552	1.1515	1.1621
tumor-A-Jun-2019	gain	116	chr18	158449	14852729	14694281	p11.32p11.21	3.8233	3.807	3.8418
tumor-A-Jun-2019	loss	88	chr14	74326992	86090091	11763100	q24.3q31.3	1.1545	1.1483	1.1587
tumor-A-Jun-2019	loss	205	chr5	79733325	123973849	44240525	q14.1q23.2	1.3682	1.3618	1.377
tumor-A-Jun-2019	loss	122	chr1	5987459	12888773	6901315	p36.31p36.21	1.1786	1.1727	1.1859
tumor-A-Jun-2019	loss	160	chr5	60240509	79733324	19492816	q12.1q14.1	1.5339	1.5258	1.5405
tumor-A-Jun-2019	gain	106	chr1	861072	5969523	5108452	p36.33p36.31	3.0759	3.0619	3.0942
tumor-A-Jun-2019	gain	37	chr1	121116437	145368934	24252498	p11.2q21.1	2.7728	2.7478	2.8062
tumor-A-Jun-2019	gain	16	chr14	19377344	20296357	919014	q11.2	2.3845	2.3168	2.469
tumor-A-Jun-2019	gain	8	chr12	11174021	11286633	112613	p13.2	2.0068	1.9197	2.1123
tumor-A-Jun-2019	loss	1	chr15	42977064	42985063	8000	q15.2	1.1922	1.1484	1.2691
tumor-A-Jun-2019	loss	1	chr14	74196093	74206961	10869	q24.3	1.1567	1.101	1.2223
tumor-A-Jun-2019	loss	10	chr1	12907011	13001563	94553	p36.21	0.0433	0.0399	0.0451
tumor-A-Jun-2019	loss	2	chrX	47917607	47920574	2968	p11.23	0	0	0
tumor-A-Jun-2019	loss	295	chr8	190646	30242924	30052279	p23.3p12	1.8007	1.7915	1.8084
tumor-A-Jun-2019	loss	401	chr17	5757	18287151	18281395	p13.3p11.2	1.8082	1.8023	1.8157
tumor-A-Jun-2019	loss	634	chr11	192850	56128923	55936074	p15.5q12.1	1.7992	1.7873	1.8075
tumor-A-Jun-2019	loss	2	chr3	75785779	75787778	2000	p12.3	0.0096	0.0083	0.0105
tumor-A-Jun-2019	loss	6	chr17	18291283	18395935	104653	p11.2	1.3259	1.2337	1.4274
tumor-B-Jun-2019	loss	395	chr12	39047434	62261456	23214023	q12q14.1	1.1898	1.1869	1.1947
tumor-B-Jun-2019	gain	1331	chr1	145414532	249212550	103798019	q21.1q44	2.7593	2.7489	2.7681
tumor-B-Jun-2019	loss	179	chr14	54866362	74194477	19328116	q22.2q24.3	1.1794	1.1731	1.1846
tumor-B-Jun-2019	loss	188	chr1	24424163	34401805	9977643	p36.11p35.1	1.211	1.2062	1.2178
tumor-B-Jun-2019	gain	1412	chr7	192950	158937713	158744764	p22.3q36.3	2.6255	2.6116	2.6337
tumor-B-Jun-2019	gain	314	chr18	18531095	78005481	59474387	q11.1q23	2.6095	2.5998	2.6272
tumor-B-Jun-2019	loss	244	chr15	79069536	102359139	23289604	q25.1q26.3	1.2001	1.1934	1.2064
tumor-B-Jun-2019	gain	848	chr1	34497945	121116297	86618353	p35.1p11.2	2.7527	2.7364	2.7689
tumor-B-Jun-2019	loss	445	chr5	122491328	162945619	40454292	q23.2q34	1.5062	1.5006	1.512
tumor-B-Jun-2019	loss	293	chr15	20739247	40913587	20174341	q11.2q15.1	1.1763	1.1689	1.1841
tumor-B-Jun-2019	gain	116	chr18	158449	14852729	14694281	p11.32p11.21	3.9075	3.8853	3.9292
tumor-B-Jun-2019	loss	206	chr1	13182741	24411379	11228639	p36.21p36.11	1.2163	1.2129	1.2203
tumor-B-Jun-2019	loss	88	chr14	74326992	86090091	11763100	q24.3q31.3	1.1811	1.1744	1.1864
tumor-B-Jun-2019	loss	478	chr15	40917588	79063786	38146199	q15.1q25.1	1.1926	1.1833	1.1971
tumor-B-Jun-2019	loss	213	chr5	60394569	89990456	29595888	q12.1q14.3	1.4784	1.4702	1.4894
tumor-B-Jun-2019	loss	122	chr1	5987459	12888773	6901315	p36.31p36.21	1.2146	1.2079	1.2195
tumor-B-Jun-2019	gain	106	chr1	861072	5969523	5108452	p36.33p36.31	3.0404	3.0221	3.0619

tumor-B-Jun-2019	gain	37	chr1	121116437	145368934	24252498	p11.2q21.1	2.77	2.7297	2.798
tumor-B-Jun-2019	loss	147	chr5	89992504	122435906	32443403	q14.3q23.2	1.3518	1.3437	1.3563
tumor-B-Jun-2019	gain	16	chr14	19377344	20296357	919014	q11.2	2.3562	2.2822	2.4738
tumor-B-Jun-2019	gain	8	chr12	11174021	11286633	112613	p13.2	1.9849	1.8989	2.0369
tumor-B-Jun-2019	loss	1	chr14	74196093	74206961	10869	q24.3	1.1603	0	1.2757
tumor-B-Jun-2019	loss	1	chr15	79063787	79069433	5647	q25.1	1.1365	1.0675	1.1912
tumor-B-Jun-2019	loss	1	chr15	40913588	40917587	4000	q15.1	1.1748	1.1129	1.2248
tumor-B-Jun-2019	loss	10	chr1	12907011	13001563	94553	p36.21	0.1924	0.1692	0.687
tumor-B-Jun-2019	loss	1	chr1	24412884	24422226	9343	p36.11	1.1673	1.122	1.2056
tumor-B-Jun-2019	loss	2	chrX	47917607	47920574	2968	p11.23	0.0012	0.0011	0.0014
tumor-B-Jun-2019	loss	3	chr2	131413872	131487474	73603	q21.1	1.6924	1.5345	1.9158
tumor-B-Jun-2019	loss	295	chr8	190646	30242924	30052279	p23.3p12	1.8051	1.7991	1.8129
tumor-B-Jun-2019	loss	2	chr3	75785779	75787778	2000	p12.3	0.0116	0.0101	0.0133

Table S2: GATK4 somatic gene variant analysis; related to Figure 1: sCNV segmentation profiles of 2019 specimens as determined by the GATK4 somatic CNV algorithm.

Table S3

chrom	pos	ref	alt	gene symbol	transcript	protein change
chr1	6947717	C	T	CAMTA1	NM_001242701.1	p.Leu80Phe
chr1	14106394	A	ACTC	PRDM2	NM_012231.4	p.Pro703dup
chr1	14143003	A	G	PRDM2	NM_001135610.1	p.Gln198Arg
chr1	15832543	T	C	CASP9	NM_001229.4	p.Gln221Arg
chr1	15850613	G	A	CASP9	NM_001229.4	p.Ala28Val
chr1	17991052	T	C	ARHGEF10L	NM_018125.3	p.Trp991Arg
chr1	18023690	A	G	ARHGEF10L	NM_018125.3	p.Ile1219Val
chr1	23885498	T	C	ID3	NM_002167.4	p.Thr105Ala
chr5	68695940	T	G	RAD17	NM_133339.2	p.Leu557Arg
chr5	112176756	T	A	APC	NM_000038.5	p.Val1822Asp
chr12	49447416	G	C	KMT2D	NM_003482.3	p.Arg228Gly
chr15	40477831	G	A	BUB1B	NM_001211.5	p.Arg349Gln
chr15	40898643	G	C	KNL1	NM_170589.4	p.Arg43Thr
chr15	40903684	A	G	KNL1	NM_170589.4	p.Thr113Ala
chr15	40913840	G	T	KNL1	NM_170589.4	p.Ala486Ser
chr15	40914177	T	C	KNL1	NM_170589.4	p.Met598Thr
chr15	40915190	A	G	KNL1	NM_170589.4	p.Arg936Gly
chr15	40916237	A	G	KNL1	NM_170589.4	p.Lys1285Glu
chr15	40916801	A	G	KNL1	NM_170589.4	p.Thr1473Ala
chr15	74328116	A	G	PML	NM_033239.2	p.Ser772Gly
chr15	74328206	G	C	PML	NM_033239.2	p.Ala802Pro
chr15	74336633	T	C	PML	NM_033238.2	p.Phe645Leu
chr15	89876827	T	TTGC	POLG	NM_001126131.1	p.Gln53dup

Table S3: COSMIC Cancer Gene Census analysis of germline variants; related to Figure 1: The list of germline variants in annotated tumor suppressor genes (TSGs) by COSMIC Cancer Gene Census were also examined for the loss of sCNV status to identify any bi-allelic inactivated gene candidates. The sole germline variant with gnomAD allele frequency <= 0.1% in a loss sCNV segment (KMT2D p.Arg228Gly) was found to have lost the mutant allele, not the wild type allele. We therefore did not identify any functionally relevant, bi-allelic inactivated TSGs in the 2019 specimens.

Table S4

sample	Sample Type	Median Usable Calling Depth	Median Insert Size	Num Pf Clusters	Aligned Pf Reads Count	Aligned Pf Reads-Percent	Duplication Percent
normal-blood	normal	89	313	57,234,845	114,264,257	99.82	22.17
tumor-2016	tumor	230	254	193,039,303	385,778,108	99.92	39.23
tumor-A-Jun-2019	tumor	207	273	134,992,302	269,516,884	99.83	24.83
tumor-B-Jun-2019	tumor	184	289	122,795,895	245,068,214	99.79	24.88
tumor-Oct-2019	tumor	403	282	263,307,126	524,690,393	99.63	25.8
**sample**	**Target Bases Percent_1X**	**Target Bases Percent_10X**	**Target Bases Percent_20X**	**Target Bases Percent_30X**	**Target Bases Percent_40X**	**Target Bases Percent_50X**	**Target Bases Percent_60X**
normal-blood	99.05	98.43	97.77	96.92	95.4	91.95	85.29
tumor-2016	99.47	99.29	99.09	98.79	98.34	97.69	96.78
tumor-A-Jun-2019	99.09	98.63	98.28	97.95	97.62	97.29	96.9
tumor-B-Jun-2019	99.09	98.58	98.16	97.77	97.4	96.99	96.48
tumor-Oct-2019	99.15	98.88	98.77	98.69	98.62	98.56	98.5

Table S4: **Quality control metrics; related to Figure 1:** The sequencing QC metrics of tumor and matching normal patient specimens used in the study.

Table S5

Hairpins selected to target each gene, full hairpin sequences

	gene name	miR-1 flank 5' (21nt)	Variable Passenger (21nt)	Loop (18nt)	Variable Guide (21nt)	miR-1 flank 3' (21nt)
	generic	CCATATTCAGCCTTTGAGAGT	NNNNNNNNNNNNNNNNNNNNN	TAGTTATATTCAAGCATA	NNNNNNNNNNNNNNNNNNNNN	GCGAAATCTGGCGAGACATCG
cluster 012.1	*ft*	CCATATTCAGCCTTTGAGAGT	**CTGGCTAAGTGTGGACAGAAA**	TAGTTATATTCAAGCATA	**TTTCTGTCCACACTTAGCCAG**	GCGAAATCTGGCGAGACATCG
	kug	CCATATTCAGCCTTTGAGAGT	**CAGAGGTTAAGTATAGGATAA**	TAGTTATATTCAAGCATA	**TTATCCTATACTTAACCTCTG**	GCGAAATCTGGCGAGACATCG
	Xpd	CCATATTCAGCCTTTGAGAGT	**CCCGAAGATACTCGATTTCGA**	TAGTTATATTCAAGCATA	**TCGAAATCGAGTATCTTCGGG**	GCGAAATCTGGCGAGACATCG
cluster 012.2	*ft*	CCATATTCAGCCTTTGAGAGT	**AACGGGTGAGGTGAAGACCAA**	TAGTTATATTCAAGCATA	**TTGGTCTTCACCTCACCCGTT**	GCGAAATCTGGCGAGACATCG
	kug	CCATATTCAGCCTTTGAGAGT	**ATCATGGGTGAACCATCAGAA**	TAGTTATATTCAAGCATA	**TTCTGATGGTTCACCCATGAT**	GCGAAATCTGGCGAGACATCG
	Xpd	CCATATTCAGCCTTTGAGAGT	**CCAGGTGACCATTTCGTCCAA**	TAGTTATATTCAAGCATA	**TTGGACGAAATGGTCACCTGG**	GCGAAATCTGGCGAGACATCG

	gene name	
cluster 012.1	*ft*	CCATATTCAGCCTTTGAGAGT**CTGGCTAAGTGTGGACAGAAA**TAGTTATATTCAAGCATA**TTTCTGTCCACACTTAGCCAG**GCGAAATCTGGCGAGACATCG
	kug	CCATATTCAGCCTTTGAGAGT**CAGAGGTTAAGTATAGGATAA**TAGTTATATTCAAGCATA**TTATCCTATACTTAACCTCTG**GCGAAATCTGGCGAGACATCG
	Xpd	CCATATTCAGCCTTTGAGAGT**CCCGAAGATACTCGATTTCGA**TAGTTATATTCAAGCATA**TCGAAATCGAGTATCTTCGGG**GCGAAATCTGGCGAGACATCG
cluster 012.2	*ft*	CCATATTCAGCCTTTGAGAGT**AACGGGTGAGGTGAAGACCAA**TAGTTATATTCAAGCATA**TTGGTCTTCACCTCACCCGTT**GCGAAATCTGGCGAGACATCG
	kug	CCATATTCAGCCTTTGAGAGT**ATCATGGGTGAACCATCAGAA**TAGTTATATTCAAGCATA**TTCTGATGGTTCACCCATGAT**GCGAAATCTGGCGAGACATCG
	Xpd	CCATATTCAGCCTTTGAGAGT**CCAGGTGACCATTTCGTCCAA**TAGTTATATTCAAGCATA**TTGGACGAAATGGTCACCTGG**GCGAAATCTGGCGAGACATCG

Spacer sequences

spacer name	derived from	sequence
G-WAL F	vector	actctgaatagggaattgggaattgagatctgttctaga
G39.1	miR-1	agtagtgccaccaaaagttagccgcgttgtggaaaatcc
G39.2	miR-279	gagggaaatggagaacgcaaaaatcccattataatggaa
G39.4	miR-8	acaaataatgttgcaataaccagttgaaaccaatggaat
G-WAL R	vector	tagcggccgcaagaattcaggcgaga

Fully assembled cluster sequences

	GWAL-F	ft	G39.1	kug	G39.2	Xpd	G39.4	GWalR
012.1	actctgaatagggaa<br>ttgggaattgagatc<br>tgttctaga	CCATATTCAGCCTTTGAGAGTCT<br>GGCTAAGTGTGGACAGAAATAGT<br>TATATTCAAGCATATTTCTGTCCA<br>CACTTAGCCAGGCGAAATCTGGC<br>GAGACATCG	agtagtgccaccaaaagttagccgcgttg<br>tggaaaatcc	CCATATTCAGCCTTTGAGA<br>GTCAGAGGTTAAGTATAGG<br>ATAATAGTTATATTCAAGCA<br>TATTATCCTATACTTAACCT<br>CTGGCGAAATCTGGCGAGA<br>CATCG	gagggaaatggagaacgcaaaaatccc<br>attataatggaa	CCATATTCAGCCTTTGAGAGTCC<br>CGAAGATACTCGATTTCGATAGT<br>TATATTCAAGCATATCGAAATCG<br>AGTATCTTCGGGGCGAAATCTG<br>GCGAGACATCG	acaaataatgt<br>tgcaataacca<br>gttgaaaccaa<br>tggaat	tagcggccgc<br>aagaattcag<br>gcgaga
012.2	actctgaatagggaa<br>ttgggaattgagatc<br>tgttctaga	CCATATTCAGCCTTTGAGAGTAA<br>CGGGTGAGGTGAAGACCAATAG<br>TTATATTCAAGCATATTGGTCTTC<br>ACCTCACCCGTTGCGAAATCTGG<br>CGAGACATCG	agtagtgccaccaaaagttagccgcgttg<br>tggaaaatcc	CCATATTCAGCCTTTGAGA<br>GTATCATGGGTGAACCATC<br>AGAATAGTTATATTCAAGC<br>ATATTCTGATGGTTCACCCA<br>TGATGCGAAATCTGGCGAG<br>ACATCG	gagggaaatggagaacgcaaaaatccc<br>attataatggaa	CCATATTCAGCCTTTGAGAGTCC<br>AGGTGACCATTTCGTCCAATAGT<br>TATATTCAAGCATATTGGACGAA<br>ATGGTCACCTGGGCGAAATCTG<br>GCGAGACATCG	acaaataatgt<br>tgcaataacca<br>gttgaaaccaa<br>tggaat	tagcggccgc<br>aagaattcag<br>gcgaga

012.1	actctgaatagggaattgggaattgagatctgttctagaCCATATTCAGCCTTTGAGAGTCTGGCTAAGTGTGGACAGAAATAGTTATATTCAAGCATATTTCTGTCCACACTTAGCCAGGCGAAATCTGGCGAGACATCGagtagtgccaccaaaagttagccgcgttg
012.2	actctgaatagggaattgggaattgagatctgttctagaCCATATTCAGCCTTTGAGAGTAACGGGTGAGGTGAAGACCAATAGTTATATTCAAGCATATTGGTCTTCACCTCACCCGTTGCGAAATCTGGCGAGACATCGagtagtgccaccaaaagttagccgcgttg

Table S5: Construction of knockdown vectors; related to Figure 1. Sequences for the hairpin, spacer and assembled knockdown vectors are listed for CPCT012.1 and CPCT012.2. The assembled vector was then placed into a transformation vector as described in Materials and Methods.

Transparent Methods

Enrollment: This work was regulated by a biorepository protocol that managed specimen acquisition, processing and inventory and a separate protocol that governed analysis of genome data, model building validation and drug screening. Patient treatment was regulated by a third protocol and a personalized treatment consent written for the therapy recommended by the multidisciplinary tumor board. All protocols and consent forms were approved by the Mount Sinai Institutional Review Board.

Whole exome sequencing: Genome assays were performed on a fresh frozen tumor specimen and whole blood collected at the time of consent to serve as a patient matched normal (*i.e.* germline) control. Protocols for sample processing, genomic assays and analysis were as previously described (Uzilov *et al.*, 2016; Bangi *et al.*, 2019).

Paired-end (2×100 nt) whole exome sequencing (WES) was carried using Illumina HiSeq 2500 or 4000 instruments. Hybridization capture was carried out using SureSelect Human All Exon V5 (Agilent) for the 2016 specimen and Twist Human Core Exome for all three 2019 specimens. Libraries for tumor and normal samples were multiplexed in a 2:1 or 3:1 ratio of tumor to normal. The same normal (blood) sample was used as the matching normal for all four tumor specimens. Full details of the resulting sequencing QC result are given in Table S4.

Alignment of de-multiplexed FASTQ files and calling of molecular variants (somatic or germline SNVs and small insertions/deletions) were carried out using a Sema4 in-house pipeline (Tigris version 2.2.0). This pipeline implemented Broad Institute's best practices for running the Genome Analysis Toolkit (GATK) version 4.0.4.0 (McKenna *et al.*, 2010; DePristo *et al.*, 2011). The pipeline was written in Workflow Description Language (WDL) and executed using the Cromwell workflow engine using the Amazon AWS Batch backend. Sequencing reads were aligned to the hg19 human reference genome (*UCSC Genome Browser Downloads*, no date) using BWA-MEM version 0.7.17 (Li, 2013), duplicate reads were marked out using Picard MarkDuplicates (*Picard*, no date), and base quality recalibration was carried out using GATK4's BaseRecalibrator, resulting in BAM files used for all further analysis. Somatic mutations were called using GATK4's Mutect2 in tumor/normal analysis mode with default settings, then filtered using GATK4's FilterMutectCalls and FilterByOrientationBias. The resulting VCFs were loaded into a custom MySQL database schema using in-house scripts and annotated using RVS (Hakenberg *et al.*, 2016) and SnpEff 4.0b (Cingolani *et al.*, 2012), using the Ensembl version 75/GRCh37 resource bundle. The germline protein-altering variant KMT2D (p.Arg228Gly) was manually reviewed in IGV (Thorvaldsdóttir, Robinson and Mesirov, 2013) to inspect supporting alignment quality. Of note, the WES sequencing hybridization capture region was different in the 2016 specimen, and we could only apply GATK4 Somatic CNV to the 2019 specimens (Figure S1).

Somatic copy number variants (sCNV) calling and analysis: All 2016 and 2019 specimens were analyzed for sCNV using saasCNV (Zhang and Hao, 2015). In addition, since we have internal normal (blood) samples from other individuals sequenced via the same hybridization capture kit (Twist Human Core Exome) similar to the three 2019 specimens, we were able to create a panel of control samples; we therefore used GATK4 somatic CNV as an alternative sCNV calling tool to confirm the sCNV in all three 2019 specimens. NOTCH1 amplification was functionally confirmed by identifying nuclear-localized protein using immunohistochemical assays on tumor sections using previously described staining and scoring protocols (Donovan *et al.*, 2009; Bangi *et al.*, 2019)**.**

Model Building and Validation: In order to generate the patient model, a previously reported multigenic Drosophila transformation vector (Ni *et al.*, 2011; Bangi *et al.*, 2019) that contains three UAS cassettes was used. Each UAS cassette in the vector contains a unique multiple cloning site (MCS) flanked by a UAS promoter and SV40 transcription terminator sequence. The coding sequence for the truncated MYB protein observed in the patient was generated by PCR from a cDNA clone of the human MYB gene using the following forward and reverse primer sequences respectively: atgcGGCCGGCCcaaaATGGCCCGAAGACCCCG and atgcTTAATTAATTACTGCAAGGGGCTCGCCA. These primers also included restriction sites for enzymes FseI and PacI to the 5` and 3` ends of the product respectively, which were used to clone the amplified product into MCS1 of the multigenic vector (Figure 1D).

For gene knockdown, our previously established synthetic short hairpin cluster design where individual short hairpins were separated by spacer sequences found 5` to well expressed endogenous microRNAs in the Drosophila genome. Criteria for short hairpin selection and the protocol for short cluster design have both been previously reported (Vert *et al.*, 2006; Ni *et al.*, 2011; Bangi *et al.*, 2019). In order to increase the likelihood of success, two synthetic clusters that target the same three genes using different short hairpin sequences were designed (012.1 and 012.2). The hairpin, spacer and final cluster sequences can be found in Table S5. Cluster synthesis was outsourced to GENEWIZ. Sequence confirmed synthetic clusters 012.1 and 012.2 were separately cloned into a UAS cassette of the multigenic vector that already contained the truncated human MYB coding sequence using XbaI and NotI enzymes using restriction sites that were appended to the 5` and 3` ends of the clusters respectively. The UAS cassette that the clusters cloned into was specifically designed for short hairpin expression (Vert *et al.*, 2006; Ni *et al.*, 2011; Bangi *et al.*, 2019). The resulting two multigenic vectors both contained the same MYBΔC transgene and a different hairpin cluster targeting the same three genes using different hairpin sequences (Table S5).

After the final vectors were sequence confirmed, transgenic flies were generated by PhiC31-mediated targeted integration (Bischof *et al.*, 2007) into the second Drosophila chromosome using the landing site *attp40*. Tranegensis was outsourced to BestGene. After transgenic lines containing the multigenic vectors were generated, a previously established UAS-Notch line on chromosome three (Matsuno *et al.*, 2002) was introduced into the multigenic vector background to generate the final patient models: *w; UAS-multigenic [UAS-MYBΔC, UAS-3sh(Xpd,ft,kug)] attp40; UAS-N/S-T, Cy, Hu, Tb.*

The efficacy of gene knock-down induced by the hairpin clusters were evaluated by qPCR analysis. Experimental animals were generated by crossing patient models containing hairpin clusters 012.1 and 012.2 to a ubiquitously expressed *gal4* line that also contains a *gal80^{ts}* (*tub-gal4, tub-gal80^{ts}*) to transiently induce transgene expression for three days during larval development. Whole larvae with genotypes 1) *tub-gal4, tub-gal80^{ts}>UAS-012.1; UAS-N* 2) *tub-gal4, tub-gal80^{ts}>UAS-012.2; UAS-N*, and 3) *tub-gal4, tub-gal80^{ts}*/+ as controls were collected for RNA extraction (three biological replicates per genotype; six larvae per replicate) and stored in 300 µl RNALater (Life Technologies). For RNA extraction, Qiagen's RNeasy Plus Kit with RNase-free DNase Set for on-column DNA digestion was used following the manufacturer's instructions. RNA concentration was measured using Qubit. For qPCR analysis, 1 µg of RNA per replicate was used to generate complementary DNA (cDNA) using the High-Capacity RNA-to-cDNA kit (Life Technologies). qPCR assays were performed using the PerfeCTa SYBR Green FastMix for IQ (VWR Scientific). To identify the best housekeeping control, a panel of 5 candidate genes (*rpl32,*

hsp83, sdha, rpl13a and cyp33) were assayed. Of these, *rpl13a* produced the most robust and consistent result across replicates and genotypes and was selected as the housekeeping control. qPCR data were analyzed using the ΔΔC(t) method as previously described (Sopko *et al.*, 2014).

Quantifying *ptc>CPCT012*: Wing discs from wandering w^- (control) and *ptc>CPCT012* L3 larvae grown at 27 °C were fixed with 4% paraformaldehyde, mounted with Vectashield plus DAPI and imaged on a Leica DM5500 Q microscope. The same exposure (220 ms, 545 ms) and gain (2.8, 6.0) was used within each channel for all images. The *ptc* region was quantified using FIJI (ImageJ, v2.1.0) and represented as a ratio of the *ptc* region to total wing disc area to account for varying sizes of each individual wing disc. An outline was drawn around (i) the *ptc* region (GFP) or (ii) the entire wing disc (DAPI) manually using the freehand selection tool to exclude extraneous signal from the peripodial membrane, trachea, etc. Areas within the outlines were measured by establishing a threshold (*adjust→threshold*), then quantifying the GFP-labeled area (*analyze→analyze particles*). Area Ratios were calculated, graphed, and analyzed by Student's t-test (Microsoft Excel).

Drug Screening: Focused FDA Library was custom-made in house using drugs individually purchased as powder from Selleck Chemicals, LC Laboratories, TOcris Bioscience or MedChemExpress depending on availability. Stock solutions were made by dissolving drugs in water or 100% dimethyl sulfoxide (DMSO) at the highest possible concentration based on solubility information provided by the manufacturers. For each drug, the highest dose with no discernible toxicity (Maximum Tolerable Dose or MTD) on wildtype animals was selected for screening. Full FDA Library used for this patient was purchased from Selleck Chemicals in a 96-well format (100 µl of 10 mM solution). Both libraries were aliquoted into 384-well plates for screening. Drug screens were conducted at a single dose for each drug for both libraries along with DMSO and no DMSO controls (8 replicates per condition for Focused FDA screens and 4 replicates per condition for the Full FDA screens)

Drug-food was prepared using the PerkinElmer automated liquid handling workstation by mixing 0.7 µl of drug from the screening plate with 700 µl of semi-defined Drosophila medium (recipe available from the Bloomington Drosophila Stock Center) in 12 mm by 75 mm round-bottom test tubes (Sarstedt). As a result, each drug was diluted 1:1000 in the food, also bringing the DMSO concentration to 0.1% .

Drug food for combination screens were prepared by first adding 0.7 µl of 18 mM tofacitinib into each tube followed by 0.7 µl of each drug in the library and finally 700 µl Drosophila medium and mixing by repeated pipetting using the PerkinElmer automated liquid handling workstation, resulting in a 1:1000 dilution of each drug in the food and a DMSO concentration of 0.2%. Drug food for hit retests were prepared by hand, using a new batch of powder drug.

Experimental animals for drug screening were generated from the following cross: *w/Y; UAS-012.2; UAS-N X w; ptc-gal4, tub-gal80^{ts}* and directly aliquoted into the drug-food tubes as embryos after the food was solidified. Crosses were set up *en masse* in cages which produced embryos for four to five consecutive days laid on apple-juice plates supplemented with fresh yeast paste. Egg lays were performed at 22 °C for 24 hours to minimize transgene expression during embryogenesis and prevent embryonic lethality or irreversible developmental defects that could not be rescued by drug feeding during larval development.

Embryos were collected from fresh apple juice plates every day and embryo suspensions were generated in an "embryo buffer" designed to minimize embryo clumping and setting while aliquoting (15% glycerol, 1% Bovine

Serum Albumin, 0,1% Tween-20 in water). 7.5-10 µl embryo suspension was added to each drug-food tube using a single-channel, variable volume multi-dispense electronic pipette.

After the embryos were aliquoted, drug tubes were transferred to 29 °C to induce transgene expression. Tubes were scored for survival to adult stage 12 days later by counting both total number of experimental pupae (EP) and those that were empty, which reflected the number of experimental adults (EA). Drugs that showed significantly higher survival to the adult stage compared to controls based on multiple t tests (PRISM software) were considered hits.
